# Rhodococcus navarretei sp. nov. and Pseudarthrobacter quantipunctorum sp. nov., two novel species with the ability to biosynthesize fluorescent nanoparticles, isolated from soil samples at Union Glacier, Antarctica

**DOI:** 10.1099/ijsem.0.006536

**Published:** 2024-10-03

**Authors:** Valentina Carrasco, Matías Vargas-Reyes, Sebastián Lagos-Moraga, Claudio Dietz-Vargas, Daniela Allendes-Ormazábal, Juan Meza-Inzunza, Fernanda Rojas-Morales, Sebastián Durán-Villegas, Felipe Valenzuela-Ibaceta, Ma. Ángeles Cabrera, José M. Pérez-Donoso

**Affiliations:** 1 Universidad Andres Bello, BioNanotechnology and Microbiology Laboratory, Center for Bioinformatics and Integrative Biology (CBIB), Facultad de Ciencias de la Vida, Av. República # 330, Santiago, Chile

**Keywords:** Antarctica, *Pseudarthrobacter*, quantum dots, *Rhodococcus*, Union Glacier

## Abstract

Two Gram-stain-positive bacterial strains, EXRC-4A-4^T^ and RC-2-3^T^, were isolated from soil samples collected at Union Glacier, Antarctica. Based on 16S rRNA gene sequence similarity, strain EXRC-4A-4^T^ was identified as belonging to the genus *Rhodococcus*, and strain RC-2-3^T^ to the genus *Pseudarthrobacter*. Further genomic analyses, including average nucleotide identity and digital DNA–DNA hybridization, suggested that these strains represent new species. Strain EXRC-4A-4^T^ exhibited growth at temperatures ranging from 4 to 28 °C (optimum between 20 and 28 °C), at pH 5.0–9.0 (optimum, pH 6.0), and in the presence of 0–5.0% NaCl (optimum between 0 and 1% NaCl). Strain RC-2-3^T^ grew at 4–28 °C (optimum growth at 28 °C), pH 6.0–10 (optimum, pH 7.0) and in the presence of 0–5.0% NaCl (optimum, 1% NaCl). The fatty acid profile of EXRC-4A-4^T^ was dominated by C_17:1 ω-7_, while that of RC-2-3^T^ was dominated by anteiso-C_15 : 0_. The draft genome sequences revealed a DNA G+C content of 64.6 mol% for EXRC-4A-4^T^ and 65.8 mol% for RC-2-3^T^. Based on this polyphasic study, EXRC-4A-4^T^ and RC-2-3^T^ represent two novel species within the genera *Rhodococcus* and *Pseudarthrobacter*, respectively. We propose the names *Rhodococcus navarretei* sp. nov. and *Pseudarthrobacter quantipunctorum* sp. nov. The type strains are *Rhodococcus navarretei* EXRC-4A-4^T^ and *Pseudarthrobacter quantipunctorum* RC-2-3^T^. These strains have been deposited deposited in the CChRGM and BCCM/LMG culture collections with entry numbers RGM 3539/LMG 33621 and RGM 3538/LMG 33620, respectively.

## Introduction

The genus *Rhodococcus* belongs to the family *Nocardiaceae*, order *Mycobacteriales*, class *Actinomycetia*, phylum *Actinomycetota*. It was first described by Zopf in 1891 with *Rhodococcus rhodochrous* as the type species and classified in the order *Corynebacteriales* [1)] and reestablished by Tsukamura in 1974 to accommodate six species of *Rhodococcus*: *R. aurantiacus*, *R. bronchialis*, *R. rhodochrous*, *R. roseus*, *R. rubropertinctus*, and *R. terrae* [2)]. At the time of writing, there are 55 validly published and correct names in the genus *Rhodococcus* (https://lpsn.dsmz.de/genus/rhodococcus, accessed July 2024). *Rhodococcus* species are generally aerobic, Gram-stain-positive to Gram-stain-variable, nonmotile, with a rod–coccus growth cycle [3)]. The genus is diverse and includes species with a wide range of metabolic capabilities. *Rhodococcus* genus members are found in various environments, such as dung, soil, and marine sediment, displaying diverse cell morphologies. They produce short rods, filaments with branching, or branched substrate mycelium during early growth. In the stationary phase, common formations include cocci and short rods resulting from the fragmentation of rods, filaments, and hyphae [4)]. This diverse genus encompasses around 50 validly published species, sourced from a wide array of environments such as soil and freshwater ecosystems [5)]. Notably, rhodococci have been identified as integral components of various holobionts [6)]. While a handful of strains are known to be pathogenic for humans, animals, and plants, most of the bacteria within this genus act as commensals or have been recognized as beneficial for health [7)].

In the case of the genus *Pseudarthrobacter*, it has been recently reclassified from the genus *Arthrobacter* based on distinctions in phylogenetic positioning and chemotaxonomic characteristics. These variances include differences in polar lipid profile, quinone system, and peptidoglycan type between the two groups [8,9)]. The genus *Pseudarthrobacter* belongs to the family *Micrococcaceae*, order *Micrococcales*, class *Actinomycetia*, phylum *Actinomycetota.* At the time of writing, there are only 14 validly published and correct names in the genus *Pseudarthrobacter* (https://lpsn.dsmz.de/genus/pseudarthrobacter, accessed July 2024). Members of the genus *Pseudoarthrobacter* are typically Gram-positive, non-motile, aerobic, and characterized by rod-shaped cells [10)]. *Pseudoarthrobacter* species exhibit metabolic versatility with the ability to utilize various carbon sources [11)], including some strains that have been reported to degrade complex organic compounds [9,12)]. These bacteria can be found in diverse habitats, such as soil and other environmental niches [9,10)]. Their presence in soil suggests a role in nutrient cycling and organic matter decomposition. In all the available literature and microbial databases linked to the genus *Pseudarthrobacter*, such as the LPNS (https://lpsn.dsmz.de/genus/pseudarthrobacter) and EzBioCloud servers (https://www.ezbiocloud.net/search?tn=Pseudarthrobacter), there are only 14 type strains of the genus *Pseudarthrobacter*, and only 16 genomes have been deposited [9)]. In this report, we describe strains EXRC-4A-4^T^ and RC-2-3^T^ isolated from an Antarctic soil sample from Union Glacier, which belong to the genera *Rhodoccocus* and *Pseudarthrobacter*, respectively, by determining their phenotypic properties and taxonomically characterizing them based on their 16S rRNA gene and genome sequences. Previous work in our research group elucidated that both strains showed resistance to ultraviolet (UV) light type B (UV-B) and C (UV-C), and capabilities of biosynthesizing cadmium sulphide (CdS) nanoparticles [13)], which makes them promising micro-organisms for biotechnological purposes, and highlights the importance of these less-explored environments as a source.

## Isolation and ecology

Two soil samples, GUJRC-2 and GUJRC-4A, were aseptically collected from two sites (79° 47′ 28.8″ S 82° 55′ 57.6″ W and 79° 47′ 27.8″ S 82° 55′ 46.5″ W, respectively) at Rossman Cove in Ellsworth Mountains (Union Glacier, Antarctica).

Bacterial isolation was performed as previously described [13]. Three grams of soil were resuspended in 30 ml of sterile PBS and incubated at 20 °C for 48 h to detach bacterial cells from the soil. Subsequently, 30 µl of the bacterial suspension were plated on Reasoner’s 2A (R2A) agar [14)] and incubated at 28 °C until bacterial colonies appeared, which were then isolated.

Strains EXRC-4A-4^T^ and RC-2-3^T^ were deposited into the CChRGM and BCCM/LMG collections with entry numbers RGM 3539^T^/LMG 33621^T^ and RGM 3538/LMG 33620, respectively.

## CdS nanoparticle biosynthesis

The extracellular biosynthesis of CdS nanoparticles (quantum dots) was carried out by the method described previously [15)]. Strains EXRC-4A-4^T^ and RC-2-3^T^ were grown aerobically in lysogeny broth (LB) and R2A broth at 28 °C until reaching stationary phase. Cells were harvested by centrifugation, washed with borax-citrate buffer (30 mM borax, 15 mM citrate, pH 9.3) and resuspended in the same buffer to an optical density of 0.8 at 600 nm. Cultures were supplemented with 1 mM l-cysteine and 0.1 mM CdCl_2_ and incubated at room temperature for 35 min. One millilitre aliquots were collected and centrifuged. The formation of CdS quantum dots was evaluated by exposing the supernatant to UV light (360 nm). The optical properties of the nanoparticles were analysed using a Synergy H1 microplate reader (BioTek Instrument Inc.). Absorbance spectra were measured between 300 and 700 nm. Fluorescence spectra were measured between 390 and 700 nm, with 360 nm excitation.

**Fig. 1.**
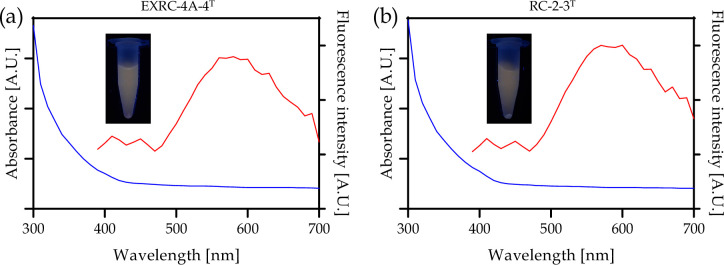
Absorbance (blue) and fluorescence emission (red) spectra of extracellular CdS nanoparticles synthesized by EXRC-4A-4^T^ (a) and RC-2-3^T^ (b) strains. Insets of the graphs show tubes containing cell supernatants, exposed to UV light (365 nm), after biosynthesis.

After incubation of the cultures and exposure to UV light, cell supernatant emitted fluorescence, which indicates the extracellular formation of nanoparticles (Fig. 1, insets). Absorbance spectra of nanoparticles for both strains show small peaks around 400 nm (Fig. 1, blue), while fluorescence emission spectra show broad peaks between 550 and 600 nm (Fig. 1, red). These optical properties are characteristic of CdS nanoparticles, which further indicates the ability of these strains to biosynthesize fluorescent nanoparticles [15,16])

## 16S rRNA gene phylogeny

Extraction of genomic DNA was carried out with the Quick-DNA Faecal/Soil Microbe Miniprep Kit (Zymo Research) following the manufacturer’s instructions, and amplification of nearly full-length 16S rRNA gene fragments was performed as previously described [16)]. The resulting amplicons were sequenced at AUSTRAL-omics (Valdivia, Chile). Sequences were further compared to other type strain sequences at the EzBioCloud server, and pairwise sequence similarity was calculated using a global alignment technique [17)].

Phylogenetic relationships between strains EXRC-4A-4^T^ and RC-2-3^T^, and their closely related strains of genus *Rhodococcus* and *Pseudarthrobacter* were analysed through a phylogenetic tree reconstructed with the neighbour-joining method [18,19)] using the mega 11.0 software package [20)]. Phylogenetic trees inferred with the maximum-likelihood [21)] and maximum-parsimony algorithms were constructed analogously. In all cases, branch robustness was assessed by Bootstrap analyses with 1000 replicates [21)].

**Fig. 2.**
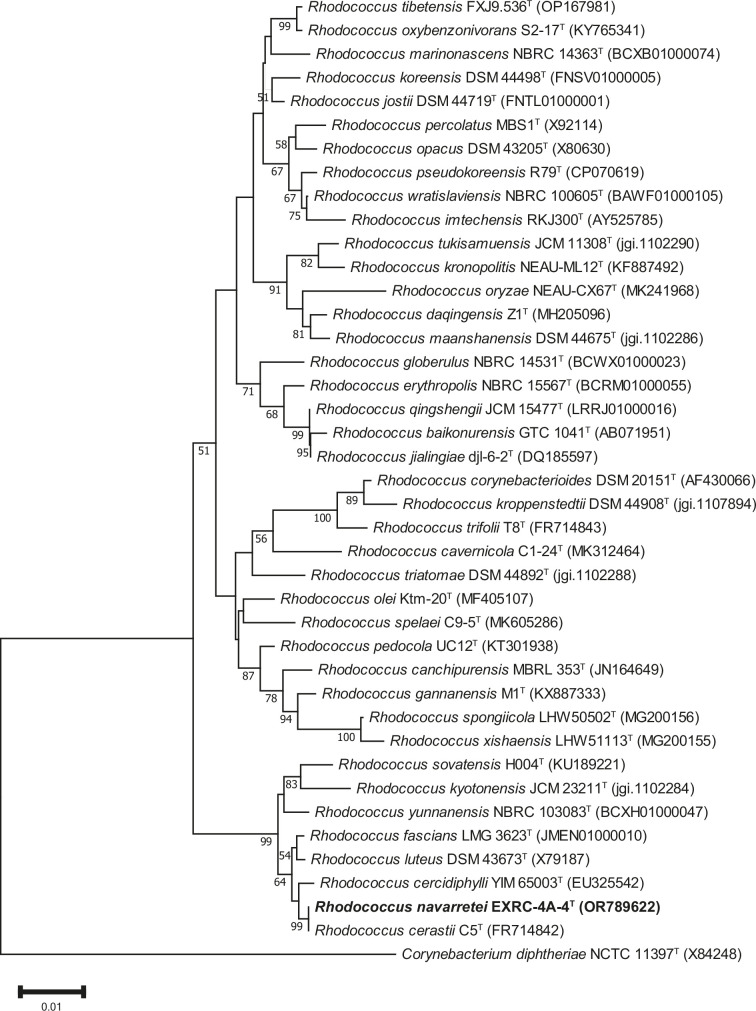
Neighbour-joining tree reconstructed with mega 11.0 based on 16S rRNA gene sequences, showing the phylogenetic relationship between strain EXRC-4A-4^T^ (highlighted in bold) and its closest related taxa. Bootstrap values were based on 1000 replicates; only values ≥50 % are shown. The bar represents 0.01 substitutions per nucleotide position. *Corynebacterium diphtheriae* NCTC 11397^T^ was used as the outgroup.

Analysis of a near-complete 16S rRNA gene sequence of strain EXRC-4A-4^T^ in the EzBioCloud server revealed that it showed high similarity to *Rhodococcus cerastii* IEGM 1327^T^ (100%), *Rhodococcus cercidiphylli* IEGM 1322^T^ (99.51%) and *Rhodococcus fascians* LMG 3623^T^ (99.17%) (Table 1). The relationship of strain EXRC-4A-4^T^ with close species visualized in the neighbour-joining tree shows a cluster with R. *cerastii* IEGM 1327^T^ and *R. cercidiphylli* IEGM 1322^T^ (Fig. 2). Phylogenetic trees reconstructed with the maximum-likelihood (Fig. S1, available in the online version of this article) and maximum-parsimony (Fig. S2) algorithms show the close relationship between strain EXRC-4A-4^T^ and *R. cerastii* IEGM 1327^T^, as well. These results suggest that strain EXRC-4A-4^T^ represents a species of the genus *Rhodococcus*.

**Table 1.** Comparison of genome statistics among closest species of *Rhodococcus*

Organism	16S rRNA gene similarity (%)*	ANI(%)*	dDDH(%)*	G+**C**(mol%)	Genome size(bp)	Assembly accession no.
*Rhodococcus navarretei* EXRC-4A-4^T^	100	100	100	64.6	5 318 342	GCA_038069675
*Rhodococcus cerastii* IEGM 1327^T^	100	89	38.8	64.8	5 228 030	GCA_033042195
*Rhodococcus cercidiphylli* IEGM 1322^T^	99.51	89	37.4	64.8	5 505 914	GCA_033042225
*Rhodococcus fascians* LMG 3623^T^	99.17	85	28.2	64.4	5 769 117	GCA_001894785
*Rhodococcus yunnanensis* NBRC 103083^T^	99.10	80	20.2	63.9	6 373 933	GCA_001895005
*Rhodococcus kyotonensis* JCM 23211^T^	98.19	80	20.2	64.2	6 309 908	GCA_900188125
*Rhodococcus erythropolis* R138^T^	96.94	78	20.3	62.3	6 806 506	GCA 000696675
*Rhodococcus kroppenstedtii* DSM 44908^T^	96.45	79	20.2	70.1	4 082 826	GCA_900111805
*Rhodococcus rhodnii* ATCC 35071^T^	95.86	78	20.2	69.7	4 458 924	GCA_008011915
*Rhodococcus qingshengii* JCM 15477^T^	97.15	79	20.1	62.4	7 261 179	GCA_023221595
*Rhodococcus corynebacterioides* NBRC 14404^T^	96.46	79	20.0	70.2	3900 442	GCA_001894765

*Percentages of 16S rRNA gene similarity, average nucleotide identity (ANI), and digital DNA–DNA hybridization (dDDH) when compared with *Rhodococcus navarretei* EXRC-4A-4T.

**Table 2.** Comparison of genome statistics among closest species of *Rhodococcus*

Organism	16S rRNA gene similarity (%)*	ANI(%)*	dDDH(%)*	G+**C**(mol%)	Genome size(bp)	Assembly accession no.
*Pseudarthrobacterquantipunctorum* RC-2-3^T^	100	100	100	65.8	4 301 960	CP148033.1
*Pseudarthrobacter polychromogenes* DSM 20136^T^	99.65	91	43.6	65.8	4 345 645	GCA_014644495
*Pseudarthrobacter scleromae* YH-2001^T^	99.72	90	41.8	66.0	4 195 919	GCA_014644515
*Pseudarthrobacter oxydans* RH60^T^	99.86	86	29.6	65.7	4 440 103	GCA_034258515
*Pseudarthrobacter phenanthrenivorans* Sphe3^T^	99.03	85	27.9	65.4	4 535 320	GCA_000189535
*Pseudarthrobacter siccitolerans* 4 J27^T^	99.10	84	25.6	65.1	4 765 168	GCA_001046895
*Arthrobacter nitrophenolicus* SJCon^T^	97.85	83	25.1	66.2	4 378 911	GCA_000332815
*Arthrobacter bangladeshi* MAHUQ-56^T^	97.03	83	24.5	66.3	4 561 718	GCA_019710585
*Pseudarthrobacter equi* IMMIB L-1606^T^	97.92	83	24.3	66.1	4 459 078	GCA_900105535
*Pseudarthrobacter chlorophenolicus* A6^T^	98.54	82	24.2	65.9	5 020 975	GCA_000022025
*Pseudarthrobacter defluvii* E5^T^	98.33	83	24.2	65.8	4 513 380	GCA_030323865

*Percentage of 16S rRNA gene similarity, average nucleotide identity (ANI), digital DNA–DNA hybridization (dDDH) when compared with *Pseudarthrobacter quantipunctorum* RC-2-3T.

**Fig. 3.**
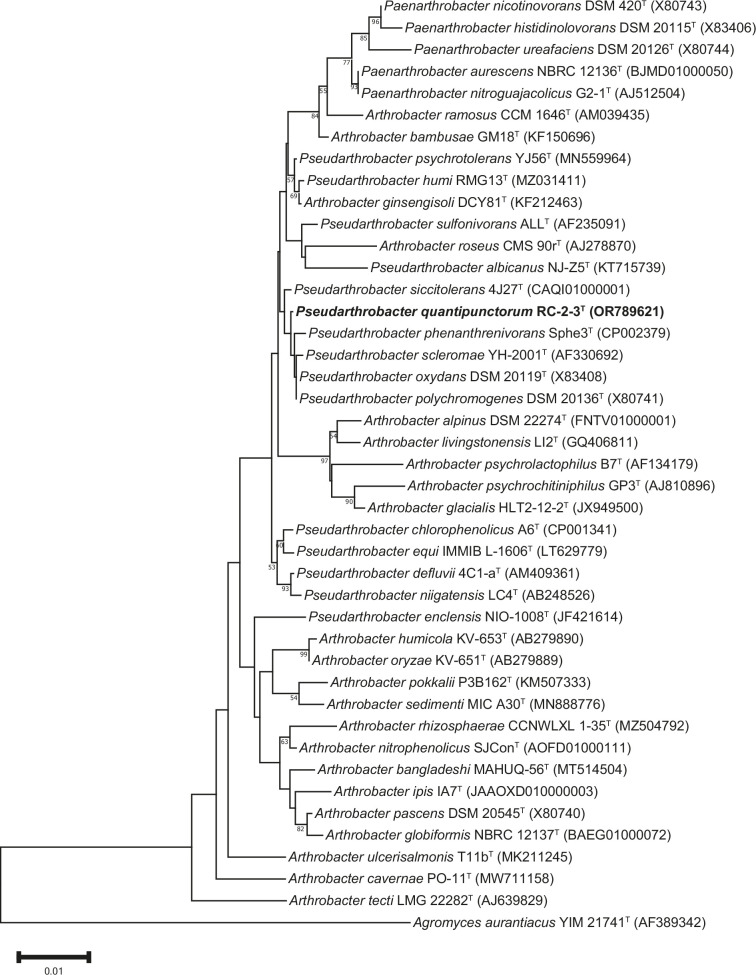
Neighbour-joining tree reconstructed with mega 11.0 based on 16S rRNA gene sequences, showing the phylogenetic relationship between strain RC-2-3^T^ (highlighted in bold) and its closest related taxa. Bootstrap values were based on 1000 replicates; only values ≥50 % are shown. The bar represents 0.01 substitutions per nucleotide position. *Agromyces aurantiacus* YIM 21741^T^ was used as the outgroup.

Analysis of strain RC-2-3^T^ revealed that the strain showed high similarity to *Pseudarthrobacter oxydans* DSM 20119^T^ (99.86%), *Pseudarthrobacter scleromae* YH-2001^T^ (99.72%) and *Pseudarthrobacter polychromogenes* DSM 20136^T^ (99.65%) (Table 1). The neighbour-joining tree shows affiliations with species of the genus *Pseudarthrobacter* and a cluster with *P. phenanthrenivorans* Sphe3^T^, *P. scleromae* YH-2001^T^, *P. oxydans* DSM 20119^T^ and *P. polychromogenes* DSM 20136^T^ (Fig. 3). Phylogenetic trees reconstructed with the maximum-likelihood (Fig. S3) and maximum-parsimony (Fig. S4) algorithms show the same cluster between the above-mentioned species. These results suggest that strain RC-2-3^T^ represents a species of the genus *Pseudarthrobacter*.

## Genome features

Whole genomes of strains RC-2-3^T^ and EXRC-4A-4^T^ were sequenced using a hybrid approach, which included Illumina NovaSeq 6000 platform producing 2×150 bp paired-end reads and Oxford Nanopore Technology (ONT) long-read sequencing (SeqCenter). According to the sequencing facility’s protocol, ONT raw sequences were trimmed with Porechop (version 0.2.3_seqan2.1.1), while Illumina sequence demultiplexing, quality control and adapter trimming were performed with bcl-convert1 (version 4.1.5). *De novo* genome assemblies were constructed from ONT reads using Flye2 (version 2.9.1) and polished with Pilon3 (version 1.23) using Illumina reads. This resulted in an assembly of three contigs comprising 5 318 342 bp for EXRC-4A-4^T^, and a complete genome assembly of 4 301 960 bp for RC-2-3^T^ (Tables1  2). Genomes of strains RC-2-3^T^ and EXRC-4A-4^T^ were annotated by the NCBI Prokaryotic Genome Annotation Pipeline with 3832 and 4913 total proteins, respectively [22)], and the Kyoto Encyclopedia of Genes and Genomes [23)].

**Fig. 4.**
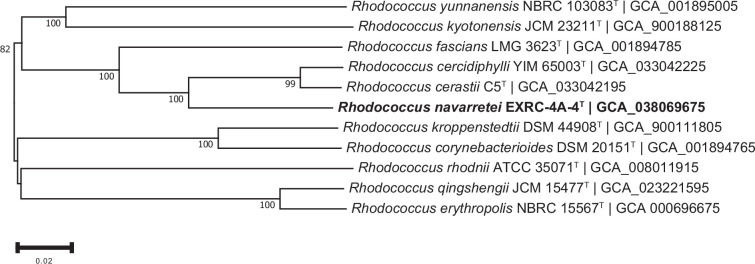
Tree inferred with FastME 2.1.6.1 from GBDP distances calculated from genome sequences. The branch lengths are scaled in terms of GBDP distance formula d5. The numbers above branches are GBDP pseudo-bootstrap support values > 60 % from 100 replications, with an average branch support of 90.6 %. The tree was rooted at the midpoint.

**Fig. 5.**
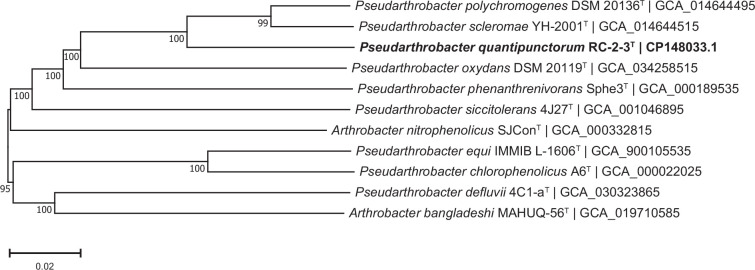
Tree inferred with FastME 2.1.6.1 from GBDP distances calculated from genome sequences. The branch lengths are scaled in terms of GBDP distance formula d5. The numbers above branches are GBDP pseudo-bootstrap support values > 60 % from 100 replications, with an average branch support of 99.3 %. The tree was rooted at the midpoint.

For the phylogenomic inference, all pairwise comparisons among the set of genomes were conducted using genome blast distance phylogeny (GBDP) and accurate intergenomic distances inferred under the algorithm 'trimming' and distance formula d5 [24)]. The resulting intergenomic distances were used to infer a balanced minimum-evolution tree with branch support via FastME 2.1.6.1, including SPR postprocessing [25)]. Branch support was inferred from 100 pseudo-bootstrap replicates each. The trees were rooted at the midpoint [26)]. The resulting phylogenomic trees showed that strain EXRC-4A-4^T^ falls within a clade with *R. cercidiphylli* YIM 65003^T^ and *R. cerastii* C5^T^ (Fig. 4), while strain RC-2-3^T^ belongs to a clade that includes *P. scleromae* YH-2001^T^ and *P. polychromogenes* DSM 20136^T^(Fig. 5).

Pairwise Digital DNA–DNA hybridization (dDDH) values between whole-genome sequences of strains RC-2-3^T^ and EXRC-4A-4^T^ and their closest related strains were determined by the Type (Strain) Genome Server (TYGS), using formula 2 (https://tygs.dsmz.de/) [24,27]Meier-Kolthoff and Göker 2019). The average nucleotide identity (ANI) values were calculated from the whole-genome sequences using the ANI calculator available at http://enve-omics.gatech.edu/(Rodriguez-R and Konstantinidis 2016)(Rodriguez-R and Konstantinidis 2016)[28].

The dDDH values between strain EXRC-4A-4^T^ and the related species ranged from 20.0 to 38.8%, while ANI values ranged from 79 to 89% (Table 1). For strain RC-2-3^T^, the dDDH values ranged from 24.2 to 43.6%, while ANI values ranged from 83 to 91% (Table 2).

For both strains, values of dDDH and ANI are all below the recommended threshold values for species limits, which are 70 and 95% for dDDH and ANI, respectively [29)]. This indicates that both strains EXRC-4A-4^T^ and RC-2-3^T^ represent new species of their respective genera.

## Physiology and chemotaxonomy

The colony phenotypes of strains EXRC-4A-4^T^ and RC-2-3^T^ were observed on tryptic soy agar (TSA) medium after incubation at 28 °C for 48 h. Cell morphology of both strains was analysed using TEM. The strains were grown in tryptic soy broth (TSB) medium at 28 °C for 16 h, then mounted on formvar/carbon-coated copper grids and negatively stained with uranyl acetate. Cells were visualized with a Talos F200C G2 microscope (Thermo Fisher Scientific), operated at 200 kV. Changes in cell morphology and Gram staining were monitored in TSB medium, with strains grown at 28 °C and samples analysed every 24 h. An MF606 microscope (BW Optics and Instrument Co) was used to visualize cells. The motility of cells was evaluated on soft TSA after growth at 28 °C for 4 days. Traditional solid media including lysogeny broth (LB) agar, R2A agar, TSA (Becton, Dickinson and Company), MacConkey agar (Becton, Dickinson and Company) and nutrient agar (NA; HiMedia) were used for growth determination at 28 °C for 4 days. Oxidase activity was assayed colorimetrically using TestOxidase reagent (Pro-Lab Diagnostics). Catalase activity was assayed based on bubble production after the addition of 3% (v/v) hydrogen peroxide (H_2_O_2_). Bacterial growth in different pH values was analysed using TSB adjusted to pH 4–10 at intervals of 1 pH unit, by the addition of HCl or NaOH. Also, different NaCl concentrations in the range of 0–5% in LB medium were used to determine the growth by measuring optical density at 600 nm at 28 °C for 3 days. Lastly, bacterial growth was examined on R2A agar medium at 4, 10, and 28 °C for 7 days. Metabolic activities and utilization of carbon compounds as the sole carbon source were determined using API 20NE and API ZYM commercial kits according to the manufacturer’s instructions (bioMérieux). Respiratory quinones were extracted as described by Albuquerque *et al*. [30)] by lyophilizing the isolates after being cultured for 24 h on TSA medium. Lyophilized cells were resuspended in 3 ml of a mixture of hexane and methanol (1 : 2) and stirred for 30 min. After stirring, the samples were cooled in ice for 30 min and 3 ml cold hexane and 2 ml NaCl 0.3% were added. For phase separation, the samples were centrifuged at 2000 *g* for 5 min and the upper phase was stored in an amber glass vial. All stages were carried out in the absence of oxygen by flushing with nitrogen. The solvent of both samples was completely evaporated using nitrogen and the samples were resuspended in 50 µl hexane. To identify whether the main quinones were menaquinones or ubiquinones, the samples were placed on a TLC Gel 60F254 plate, an 85 : 15 mixture of hexane and diethyl ether was used as mobile phase and UV-C light was used to identify the separated signals. For fatty acid extraction, strains EXRC-4A-4^T^ and RC-2-3^T^ were grown on TSA medium and a loopful of culture was used for extraction through methylation and liquid extraction of their fatty acid methyl esters (FAMEs) [31,32)]. FAMEs were analysed using a YL GC/MS 6900 system (YL Instruments). Mass spectrometer was set at an ionization energy of 70 eV, transfer line, oven, and inlet temperatures were set as described by Li *et al.* [33)]. Fatty acids were identified through their MS m/z spectra using YL Clarity software and the NIST 2008 libraries.

**Fig. 6.**
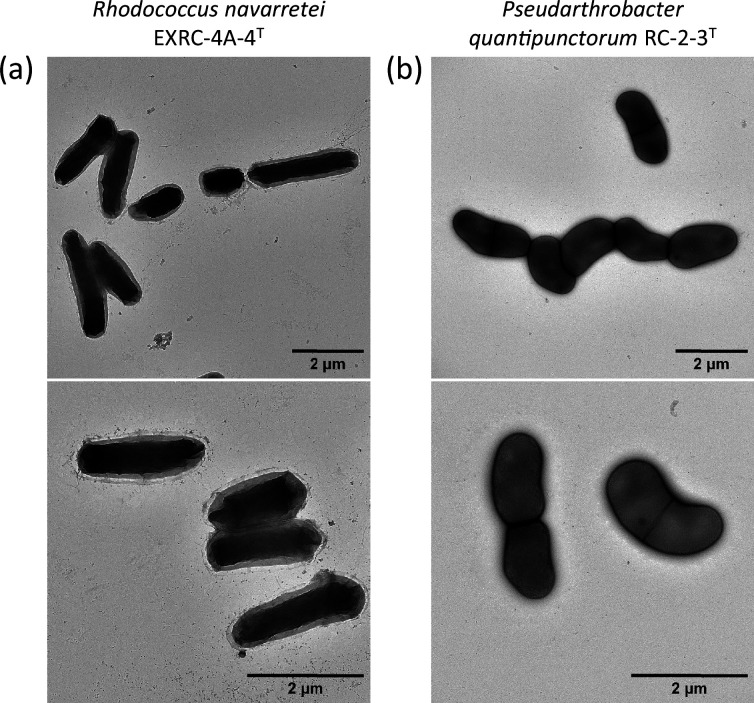
Transmission electron microscopy images of strains EXRC-4A-4^T^ (a) and RC-2-3^T^ (b) after growth in TSB medium at 28°C for 16 h.

Strain EXRC-4A-4^T^ colonies were orange, circular, 3.5 mm in diameter, and had a raised elevation after growth on TSA medium at 28 °C for 48 h. Cells were rod-shaped, 1.3–3.1 µm long and 0.7–0.9 µm wide after growth on TSA medium at 28 °C for 16 h (Fig. 6a). Cells were covered by a capsule-like structure, which, according to some reports, could be fibres of exopolymeric substances or glycocalyx [34,35)]. Cells changed from a needle-like rod morphology after 24 h of growth to smaller rods, including some coccoid cells at 120 h of growth (Fig. S5), consistent with reports of other *Rhodococcus* species [36,38)]. The strain was positive for the oxidase test and negative for the catalase test. The two predominant fatty acids of strain EXRC-4A-4^T^ were derivatives of palmitic acid, identified as C_16:1 ω-7_ and C_16 : 0_, representing 32.7 and 13.6% of the total FAMEs in EXRC-4A-4^T^. Both FAMEs are among the most represented fatty acids of their closest relatives *R. cercidiphylli* [39)] and *R. cerastii* [40)]. The third and fourth most common fatty acids in EXRC-4A-4^T^ were C_17:1 ω-7_ and C_18:1 ω-9_, with the last being the second most abundant FAME on both *R. cercidiphylli* and *R. cerastii*. C_17:1 ω-7_ was not among the main components of either of its closest relatives (Table 3).

**Table 3.** Comparison of biochemical and physiological characteristics of strain EXRC-4A-4^T^ and related species of the genus *Rhodococcus*

Strain	*Rhodococcus navarretei* EXRC-4A-4^T^	*Rhodococcus cercidiphylli* YIM 65003^T^*	*Rhodococcus cerastii* C5^T†^
Isolation source	Antarctic soil sample	Leaf sample of *Cercidiphyllum japonicum*	Leaf surface of a *Cerastium holosteoides* plant
Colony colour	Orange	Orange (opaque)	Yellowish
NaCl tolerance (w/v, %)	0–5	1–9	1–6
pH tolerance	6–10	5–9	6.5–10.5
Growth temperature (°C)	4–28	4–35	15–50
Oxidase	+	−	−
Alkaline phosphatase	(+/−)	−	(+/−)
Esterase (C4)	(+/−)	−	(+/−)
Esterase Lipase (C8)	+	−	+
Lipase (C14)	(+/−)	−	(+/−)
Leucine arylamidase	+	−	+
Valine arylamidase	+	−	+
Cystine arylamidase	+	−	(+/−)
Trypsin	+	−	(+/−)
d-Chymotrypsin	−	−	−
Acid phosphatase	+	−	(+/−)
Naphthol-AS-BI-phosphohydrolase	+	−	(+/−)
d-Galactosidase	+	−	+
β-Galactosidase	+	−	+
β-Glucuronidase	−	+	(+/−)
d-Glucosidase	+	−	+
β-Glucosidase	−	+	−
*N*-Acetyl-β-glucosaminidase	−	+	−
d-Mannosidase	−	+	−
d-Fucosidase	−	+	−
Main FAMEs	C_16 : 1ω-7_ and C_16 : 0_	C_16 : 0_ and C_18 : 1ω-9_	C_16 : 0_ and C_18 : 1ω-9_

+,Positive; −, negative.; ((+/-−)), weakly positive.

*Data from Li *et al.* [39J. Li et al., 2008].

†Data from Kämpfer *et al.* [40Kämpfer et al., 2013].

In the case of strain RC-2-3^T^, colonies were white, with a circular shape, 0.5 mm in diameter and convex elevation after growth on TSA medium at 28 °C for 48 h. Cells were coccobacilli-shaped, 1.6–1.9 µm long and 0.8–1.0 µm wide (Fig. 6b). Cells did not display noticeable morphology changes during 120 h of growth, although a Gram-stain change from negative to positive was observed after 72 h of growth, with most cells being Gram-stain positive after 96 h (Fig. S5). This behaviour has been reported for the related genus *Arthrobacter* [41,42)]. The strain was negative for the oxidase test and positive for the catalase test. Fatty acids derived from pentadecanoic acid were predominant in RC-2-3^T^, with the most abundant being anteiso-C_15 : 0_, accounting for 45.6% of all FAMEs. It should be noted that anteiso-C_15 : 0_ is also the most abundant fatty acid in its closest relative, *P. polychromogenes* [10)]. The predominant FAME was followed by iso-C_15 : 0_ and C_16 : 0_, contributing 7.2 and 7.1% of all FAMEs present on RC-2-3^T^, both being also main components of its closest relatives, *P. polychromogenes* and * P*. *scleromae*Pseudarthrobacter [43)]. Although C_17_ derivatives were found in RC-2-3^T^, anteiso C_17 : 0_ a main component of *P. polychromogenes*, was not among the main components of RC-2-3^T^ (Table 4).

**Table 4.** Comparison of biochemical and physiological characteristics of strain RC-2-3^T^ and related species of the genus *Pseudarthrobacter*

Strain	*Pseudarthrobacter quantipunctorum* RC-2-3^T^	*Pseudarthrobacter polychromogenes* DSM 20136^T^*	*Pseudarthrobacter scleromae* YH-2001^T†^
Isolation source	Antarctic soil sample	Air	Scleromata of a dermatosis patient
Colony colour	White	White	White
NaCl tolerance (w/v, %)	0–5	0–7	5–9
pH tolerance	6–10	6.0–11.0	6–9
Growth temperature (°C)	4–28	10–40	10–30
Oxidase	−	+	na
Alkaline phosphatase	+	+	−
Esterase (C4)	(+/−)	(+/−)	+
Esterase lipase (C8)	(+/−)	+	+
Lipase (C14)	−	−	+
Leucine arylamidase	+	+	−
Valine arylamidase	−	+	+
Cystine arylamidase	−	−	−
Trypsin	−	−	+
d-Chymotrypsin	−	−	+
Acid phosphatase	+	+	−
Naphthol-AS-BI-phosphohydrolase	+	+	+
d-Galactosidase	+	−	+
β-Galactosidase	+	+	+
β-Glucuronidase	+	−	−
d-Glucosidase	+	−	+
β-Glucosidase	−	−	+
*N*-Acetyl-β-glucosaminidase	−	−	−
d-Mannosidase	(+/−)	+	+
d-Fucosidase	−	−	−
Main FAMEs	anteiso-C_15 : 0_ and iso-C_15 : 0_	anteiso-C_15 : 0_ and iso-C_15 : 0_	anteiso-C_15 : 0_ and iso-C_16 : 0_

+,Positive; −, negative.; (+/-−), weakly positive.; na, not available.

*Data from Huang *et al.* [43Huang et al., 2005].

†Data from Siddiqi *et al.* [45Siddiqi et al., 2014].

The respiratory quinones preparation of isolate EXRC-4A-4^T^ presented a signal with a retention factor (RF) of 0.8 while the preparation for isolate RC-2-3^T^ presented a signal with an RF of 0.7 (Fig. S6). Based on these signals, both isolates have menaquinones as their main respiratory quinones [30)]. Furthermore, genomic analyses confirmed the presence of genes involved in the menaquinone biosynthetic pathway in both genomes (Boersch et al., 2018) (Boersch et al., 2018)[44]. These results are consistent with the genera of each isolate, as menaquinones are the main quinones of the genera *Rhodococcus* [39,40)] and * Pseudarthrobacter* [43,45)].

Both strains grew on TSA, LB, R2A and NA solid media, but not on MacConkey agar. On R2A agar medium after 7 days, both strains grew better at 28 °C than 10 or 4 °C. Cells from both strains were able to grow at pH 6–10 and NaCl concentrations of 0–5 % (Tables3  4).

Detailed descriptions of the biochemical and physiological properties of both strains are recapitulated in the species description. A comparison of the differential characteristics between strains EXRC-4A-4^T^ and RC-2-3^T^ and their closely related species are shown in Tables3  4, respectively.

In conclusion, based on the genomic and phenotypic evidence presented here, including dDDH, ANI and biochemical profiles, strains EXRC-4A-4^T^ and RC-2-3^T^ should be considered to represent novel species of the genera *Rhodococcus* and *Pseudarthrobacter*, respectively, for which we propose the names *Rhodococcus navarretei* sp. nov. and *Pseudarthrobacter quantipunctorum* sp. nov., with EXRC-4A-4^T^ and RC-2-3^T^ as the type strains.

## Description of *Rhodococcus navarretei* sp. nov.

*Rhodococcus navarretei* (na.var.re’te.i. N.L. gen. n. *navarretei* named in honour of Group Commander (AD) Eduardo Navarrete Pizarro of the Chilean Air Force (FACH), who served as Chief of the Union Glacier Station during the Joint Scientific Expedition (ECA 55) from which soil samples were obtained for the isolation of the micro-organisms used in this study. Sadly, he died in the crash of the Lockheed C-130 Hercules aircraft in the Drake Passage on 9 December 2019).

Cells of strain EXRC-4A-4^T^ are Gram-positive rods, motile aerobic, 1.3–3.1 µm long and 0.7–0.9 µm wide. Colonies on TSA are orange, circular, raised and measure 3.5 mm in diameter after 2 days at 28 °C. This strain tolerates pH between 6.0 and 10.0 (optimum, pH 7.0) and NaCl concentration from 0–5%. Oxidase test is positive and catalase test is negative. In the API 20NE system, the strain is positive for urease and β-galactosidase, and weakly positive for hydrolysis of aesculin. The type strain can assimilate glucose, arabinose, mannose, mannitol, *N*-acetyl-glucosamine, maltose, potassium gluconate, adipic acid, malate and trisodium citrate. Reduction of nitrates, indole production, glucose fermentation, arginine dihydrolase and hydrolysis of gelatine are negative. In the API ZYM system, the strain is positive for esterase lipase (C8), leucine arylamidase, valine arylamidase, cystine arylamidase, trypsin, acid phosphatase, naphthol-AS-BI-phosphohydrolase, α-galactosidase, β-galactosidase and α-glucosidase; weakly positive for alkaline phosphatase, esterase (C4) and lipase (C14); negative for α-chymotrypsin, β-glucuronidase, β-glucosidase, *N*-acetyl-β-glucosaminidase, α-mannosidase and α-fucosidase. The major fatty acids are C_16 : 0_ and C_18:1 ω-9_, C_16:1 ω-7_ and C_14 : 0_. The type strain is EXRC-4A-4^T^, isolated from a soil sample from Union Glacier in the Ellsworth Mountains, Antarctica. The genomic DNA G+C content of the type strain is 64.6 mol%. The GenBank accession number for the 16S rRNA and whole genome sequences of strain EXRC-4A-4^T^ are OR789622 and JBBPCN000000000, respectively. Strain EXRC-4A-4^T^ was deposited to CChRGM and BCCM/LMG with entry numbers RGM 3539 and LMG 33621, respectively.

## Description of *Pseudarthrobacter quantipunctorum* sp. nov.

*Pseudarthrobacter quantipunctorum* (quan.ti.punc.to’rum. L. neut. n. *quantum*, as much of; L. neut. n. *punctum*, a dot; N.L. gen. pl. n. *quantipunctorum*, of quantum dots).

Cells of strain RC-2-3^T^ are Gram-positive coccobacilli, aerobic, 1.6–1.9 µm long and 0.8–1.0 µm wide. Cells are not motile. Colonies on TSA medium are white, creamy, circular, convex and measure 0.5 mm in diameter after 2 days at 28 °C. This strain tolerates pH between 6.0 to 10.0 (optimum, pH 8.0) and NaCl concentrations from 0–5%. The oxidase test is negative and the catalase test is positive. In the API 20NE system, the type strain is positive for reduction of nitrates to nitrites, hydrolysis of aesculin and β-galactosidase. The type strain can assimilate glucose, arabinose, mannose, mannitol, *N*-acetyl-glucosamine, maltose, potassium gluconate, malate and trisodium citrate. Indole production, glucose fermentation, arginine dihydrolase, urease and hydrolysis of gelatin are negative. In the API ZYM system, the type strain is positive for alkaline phosphatase, leucine arylamidase, acid phosphatase, naphthol-AS-BI-phosphohydrolase, α-galactosidase, β-galactosidase, β-glucuronidase and α-glucosidase; weakly positive for esterase (C4), esterase lipase (C8) and α-mannosidase; negative for lipase (C14), valine arylamidase, cystine arylamidase, trypsin, α-chymotrypsin, β-glucosidase, *N*-acetyl-β-glucosaminidase and α-fucosidase. The major fatty acids are anteiso-C_15 : 0_, iso-C_15 : 0_, C_15 : 0_ and C_16 : 0_. The type strain is RC-2-3^T^, isolated from a soil sample from Union Glacier in the Ellsworth Mountains, Antarctica. The genomic DNA G+C content of the type strain is 65.8 mol%. The GenBank accession number for the 16S rRNA and whole genome sequences of strain RC-2-3^T^ are OR789621 and CP148033, respectively. Strain RC-2-3^T^ was deposited to CChRGM and BCCM/LMG with entry numbers RGM 3538 and LMG 33620, respectively.

## Availability of data and materials

The datasets used and/or analysed during the current study are available from the corresponding author upon reasonable request.

## supplementary material

10.1099/ijsem.0.006536 Uncited Fig. S1.

## Abbreviations

ANI: average nucleotide identity
dDDH: digital DNA–DNA hybridization
FAME: fatty acid methyl ester
GBDP: genome blast distance phylogeny
LB: lysogeny broth
NA: nutrient agar
R2A: Reasoner’s 2A
TSA: tryptic soy agar
