# Supplementary material for: Rhodococcus navarretei sp. nov. and Pseudarthrobacter quantipunctorum sp. nov., two novel species with the ability to biosynthesize fluorescent nanoparticles, isolated from soil samples at Union Glacier, Antarctica
Source: Int J Syst Evol Microbiol. 2024 Oct 3;74(10):006536. doi: 10.1099/ijsem.0.006536 (PMC11449289; doi:10.1099/ijsem.0.006536)
Supplement: Uncited Fig. S1. [file ijsem-74-06536-s001.pdf]

Supplementary material for

Description of Rhodococcus navarretei sp. nov. and Pseudarthrobacter quantipunctorum sp. nov., two novel species with the ability to biosynthesize fluorescent nanoparticles, isolated from soil samples at Union Glacier, Antarctica

Valentina Carrasco, Matías Vargas-Reyes, Sebastián Lagos-Moraga, Claudio Dietz-Vargas, Daniela Allendes-Ormazábal, Juan Meza-Inzunza, Fernanda Rojas-Morales, Sebastián Durán-Villegas, Felipe Valenzuela-Ibaceta, Ma. Ángeles Cabrera, José M. Pérez-Donoso

Author affiliations: Universidad Andres Bello, BioNanotechnology and Microbiology Laboratory, Center for Bioinformatics and Integrative Biology (CBIB), Facultad de Ciencias de la Vida, Av. República # 330, Santiago, Chile.
*Corresponding author: e-mail jose.perez@unab.cl

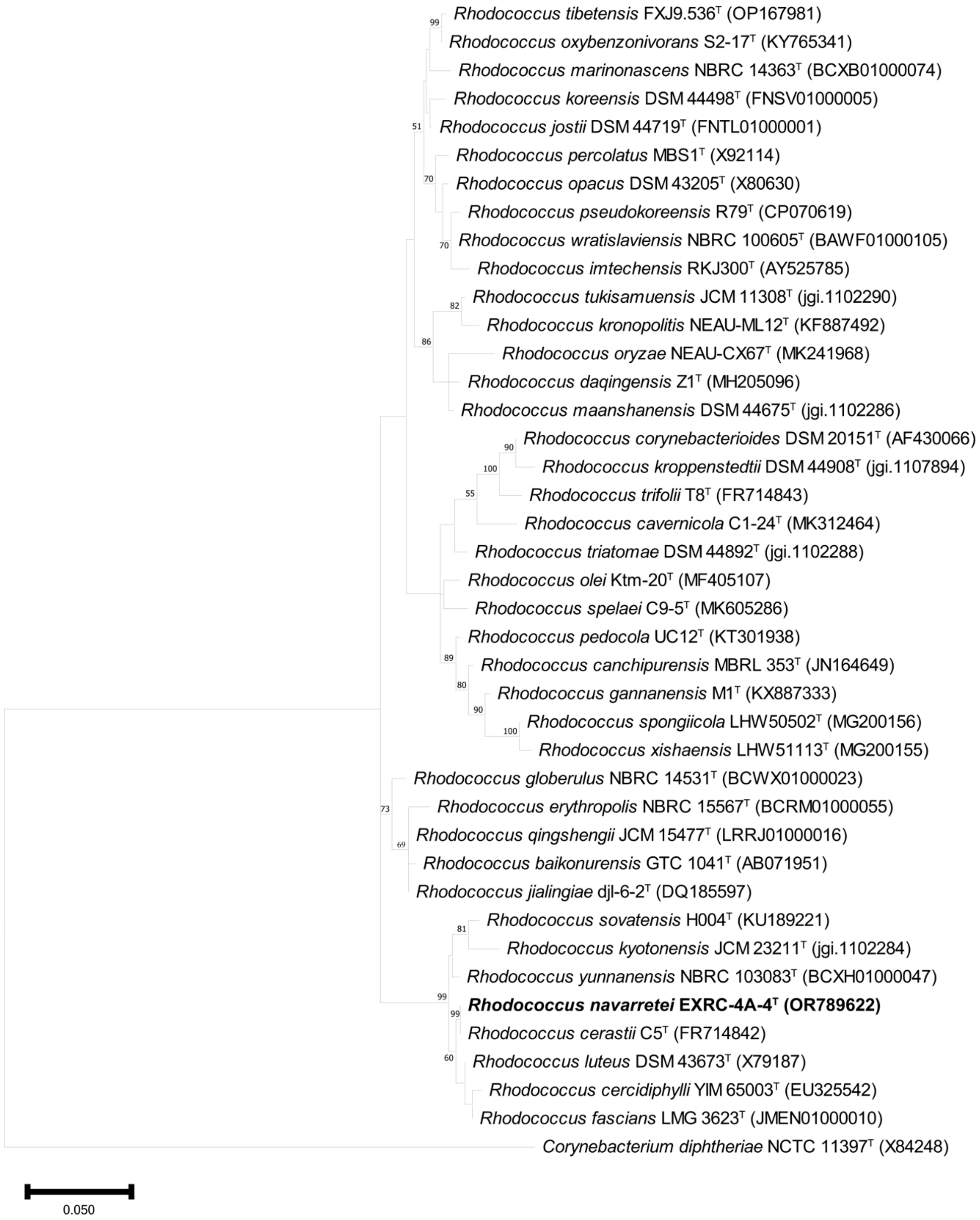
Rhodococcus tibetensis FXJ9.536^{T} (OP167981)
Rhodococcus oxybenzonivorans S2-17^{T} (KY765341)
Rhodococcus marinonascens NBRC 14363^{T} (BCXB01000074)
Rhodococcus koreensis DSM 44498^{T} (FNSV01000005)
Rhodococcus jostii DSM 44719^{T} (FNTL01000001)
Rhodococcus percolatus MBS1^{T} (X92114)
Rhodococcus opacus DSM 43205^{T} (X80630)
Rhodococcus pseudokoreensis R79^{T} (CP070619)
Rhodococcus wratislaviensis NBRC 100605^{T} (BAWF01000105)
Rhodococcus imtechensis RKJ300^{T} (AY525785)
Rhodococcus tukisamuensis JCM 11308^{T} (jgi.1102290)
Rhodococcus kronopolitis NEAU-ML12^{T} (KF887492)
Rhodococcus oryzae NEAU-CX67^{T} (MK241968)
Rhodococcus daqingensis Z1^{T} (MH205096)
Rhodococcus maanshanensis DSM 44675^{T} (jgi.1102286)
Rhodococcus corynebacterioides DSM 20151^{T} (AF430066)
Rhodococcus kroppenstedtii DSM 44908^{T} (jgi.1107894)
Rhodococcus trifolii T8^{T} (FR714843)
Rhodococcus cavernicola C1-24^{T} (MK312464)
Rhodococcus triatomae DSM 44892^{T} (jgi.1102288)
Rhodococcus olei Ktm-20^{T} (MF405107)
Rhodococcus spelaei C9-5^{T} (MK605286)
Rhodococcus pedocola UC12^{T} (KT301938)
Rhodococcus canchipurensis MBRL 353^{T} (JN164649)
Rhodococcus gannanensis M1^{T} (KX887333)
Rhodococcus spongiicola LHW50502^{T} (MG200156)
Rhodococcus xishaensis LHW51113^{T} (MG200155)
Rhodococcus globerulus NBRC 14531^{T} (BCWX01000023)
Rhodococcus erythropolis NBRC 15567^{T} (BCRM01000055)
Rhodococcus qingshengii JCM 15477^{T} (LRRJ01000016)
Rhodococcus baikonurensis GTC 1041^{T} (AB071951)
Rhodococcus jialingiae djl-6-2^{T} (DQ185597)
Rhodococcus sovatensis H004^{T} (KU189221)
Rhodococcus kyotonensis JCM 23211^{T} (jgi.1102284)
Rhodococcus yunnanensis NBRC 103083^{T} (BCXH01000047)
***Rhodococcus navarretei* EXRC-4A-4^{T} (OR789622)**
Rhodococcus cerastii C5^{T} (FR714842)
Rhodococcus luteus DSM 43673^{T} (X79187)
Rhodococcus cercidiphylli YIM 65003^{T} (EU325542)
Rhodococcus fascians LMG 3623^{T} (JMEN01000010)
Corynebacterium diphtheriae NCTC 11397^{T} (X84248)
99
51
70
70
82
86
90
100
55
89
80
90
100
73
69
81
99
99
60
0.050

Figure S1 Maximum-likelihood tree based on 16S rRNA gene sequences showing the relation of strain EXRC-4A-4^{T} and closely related species. Bootstrap values were based on 1000 replicates; only values ≥50 % are shown at the nodes. *Corynebacterium diphtheriae* NCTC 11397^{T} was used as outgroup.

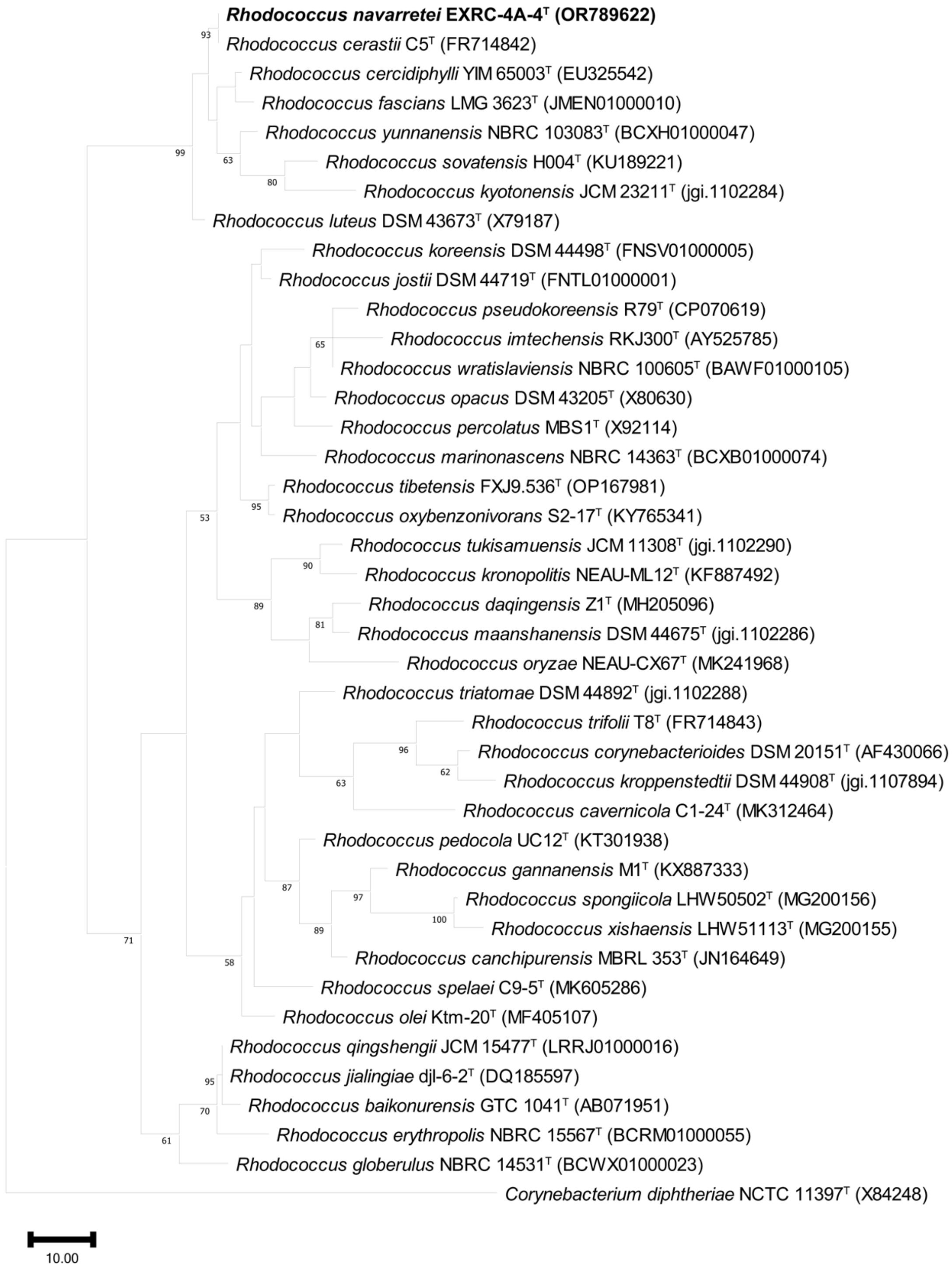
Rhodococcus navarretei EXRC-4A-4^{T} (OR789622)
Rhodococcus cerastii C5^{T} (FR714842)
Rhodococcus cercidiphylli YIM 65003^{T} (EU325542)
Rhodococcus fascians LMG 3623^{T} (JMEN01000010)
Rhodococcus yunnanensis NBRC 103083^{T} (BCXH01000047)
Rhodococcus sovatensis H004^{T} (KU189221)
Rhodococcus kyotonensis JCM 23211^{T} (jgi.1102284)
Rhodococcus luteus DSM 43673^{T} (X79187)
Rhodococcus koreensis DSM 44498^{T} (FNSV01000005)
Rhodococcus jostii DSM 44719^{T} (FNTL01000001)
Rhodococcus pseudokoreensis R79^{T} (CP070619)
Rhodococcus imtechensis RKJ300^{T} (AY525785)
Rhodococcus wratislaviensis NBRC 100605^{T} (BAWF01000105)
Rhodococcus opacus DSM 43205^{T} (X80630)
Rhodococcus percolatus MBS1^{T} (X92114)
Rhodococcus marinonascens NBRC 14363^{T} (BCXB01000074)
Rhodococcus tibetensis FXJ9.536^{T} (OP167981)
Rhodococcus oxybenzonivorans S2-17^{T} (KY765341)
Rhodococcus tukisamuensis JCM 11308^{T} (jgi.1102290)
Rhodococcus kronopolitis NEAU-ML12^{T} (KF887492)
Rhodococcus daqingensis Z1^{T} (MH205096)
Rhodococcus maanshanensis DSM 44675^{T} (jgi.1102286)
Rhodococcus oryzae NEAU-CX67^{T} (MK241968)
Rhodococcus triatomae DSM 44892^{T} (jgi.1102288)
Rhodococcus trifolii T8^{T} (FR714843)
Rhodococcus corynebacterioides DSM 20151^{T} (AF430066)
Rhodococcus kroppenstedtii DSM 44908^{T} (jgi.1107894)
Rhodococcus cavernicola C1-24^{T} (MK312464)
Rhodococcus pedocola UC12^{T} (KT301938)
Rhodococcus gannanensis M1^{T} (KX887333)
Rhodococcus spongiicola LHW50502^{T} (MG200156)
Rhodococcus xishaensis LHW51113^{T} (MG200155)
Rhodococcus canchipurensis MBRL 353^{T} (JN164649)
Rhodococcus spelaei C9-5^{T} (MK605286)
Rhodococcus olei Ktm-20^{T} (MF405107)
Rhodococcus qingshengii JCM 15477^{T} (LRRJ01000016)
Rhodococcus jialingiae djl-6-2^{T} (DQ185597)
Rhodococcus baikonurensis GTC 1041^{T} (AB071951)
Rhodococcus erythropolis NBRC 15567^{T} (BCRM01000055)
Rhodococcus globerulus NBRC 14531^{T} (BCWX01000023)
Corynebacterium diphtheriae NCTC 11397^{T} (X84248)
93
99
63
80
65
95
53
90
89
81
96
62
63
87
97
100
89
58
71
95
70
61
10.00

Figure S2. Unrooted maximum-parsimony tree based on 16S rRNA gene sequences showing the relation of strain EXRC-4A-4^T and its closest related species. Bootstrap values above 50% (based on 1000 re-samplings) are shown. *Corynebacterium diphtheriae* NCTC 11397^T was used as outgroup.

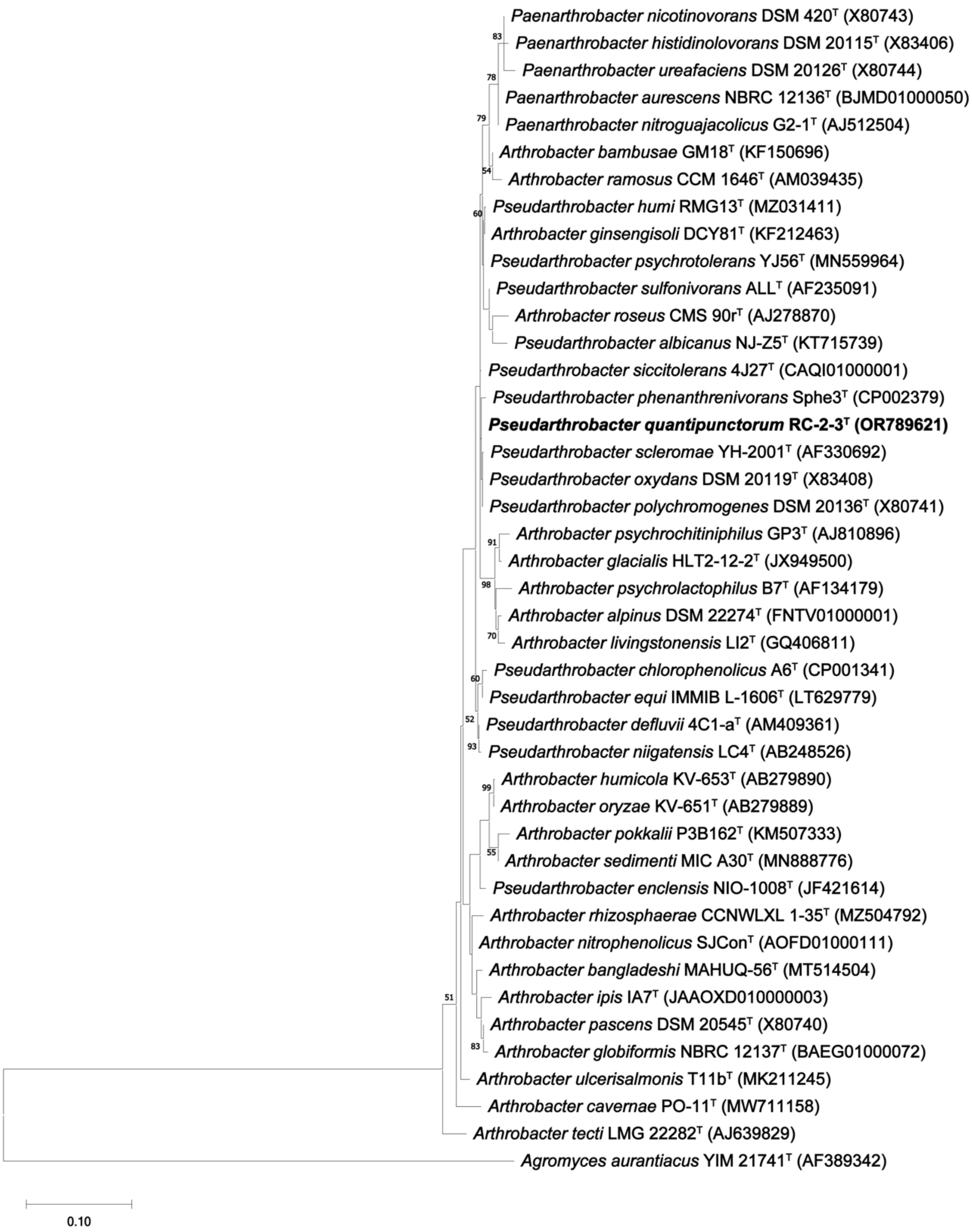

Figure S3. Maximum-likelihood tree based on 16S rRNA gene sequences showing the relation of strain RC-2-3^{T} and closely related species. Bootstrap values were based on 1000 replicates; only values ≥50 % are shown at the nodes. *Agromyces aurantiacus* YIM 21741^{T} was used as outgroup.

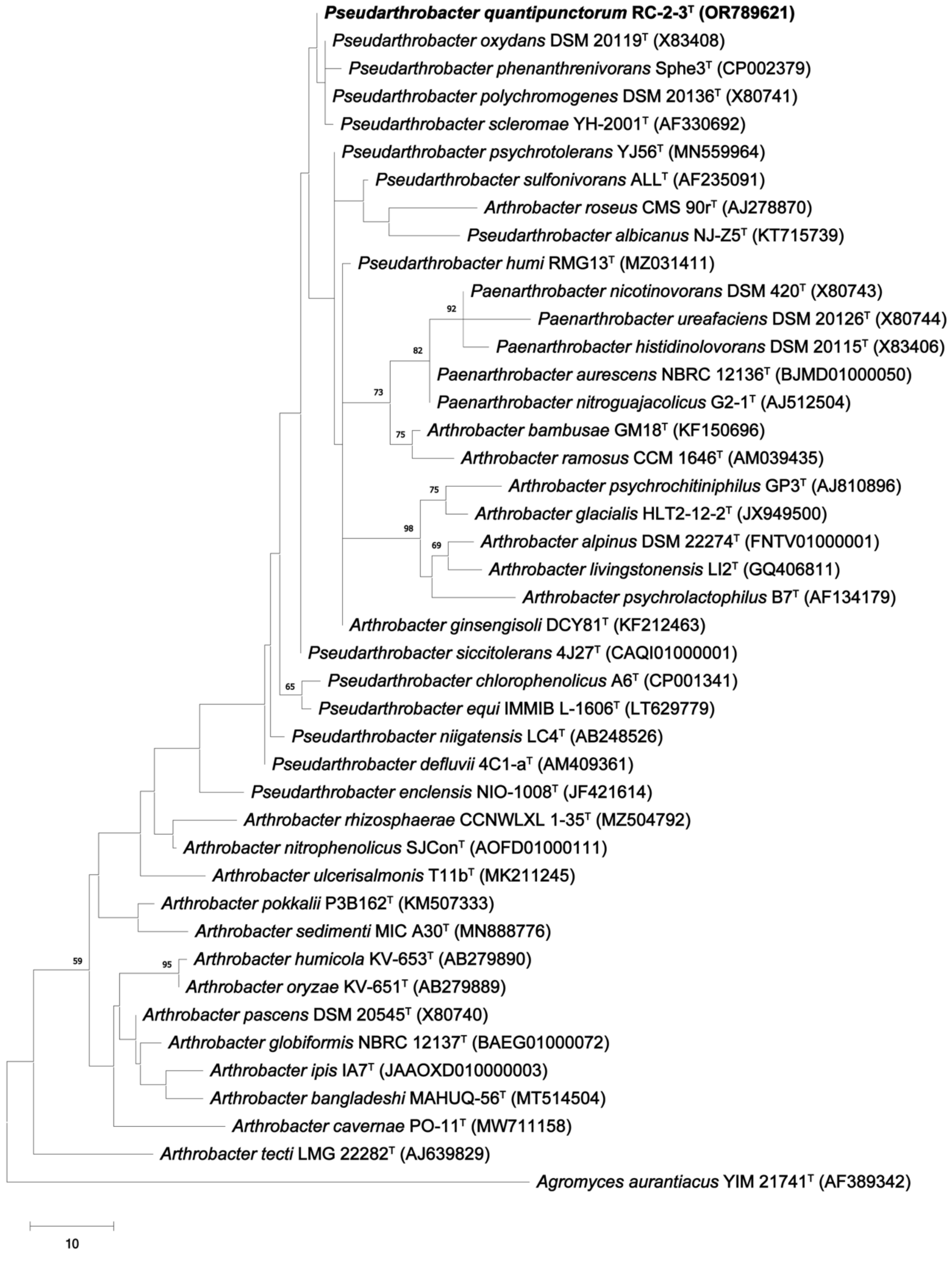

Figure S4. Unrooted maximum-parsimony tree based on 16S rRNA gene sequences showing the relation of strain RC-2-3^T and its closest related species. Bootstrap values above 50% (based on 1000 re-samplings) are shown. *Agromyces aurantiacus* YIM 21741^T was used as outgroup.

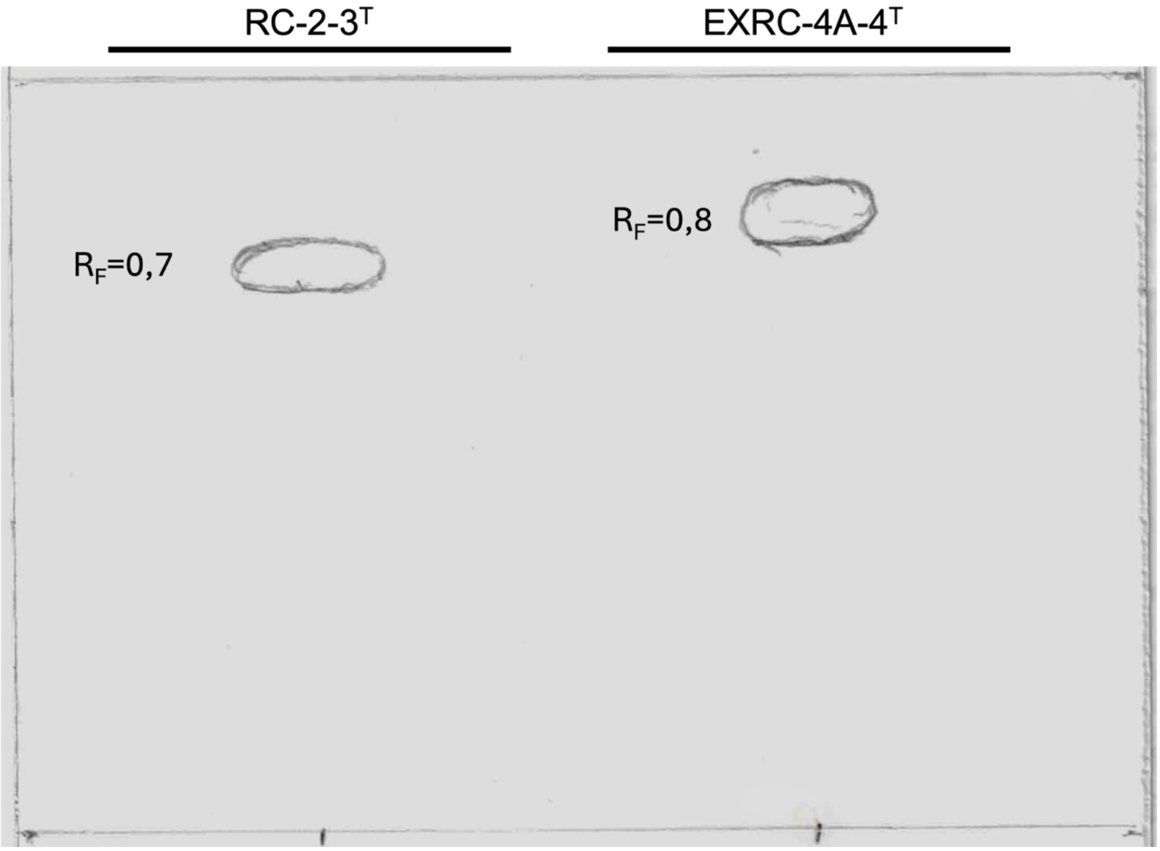

Figure S5. Thin Layer chromatography of quinones extracted from isolates RC-2-3^T and EXRC-4A-4^T.

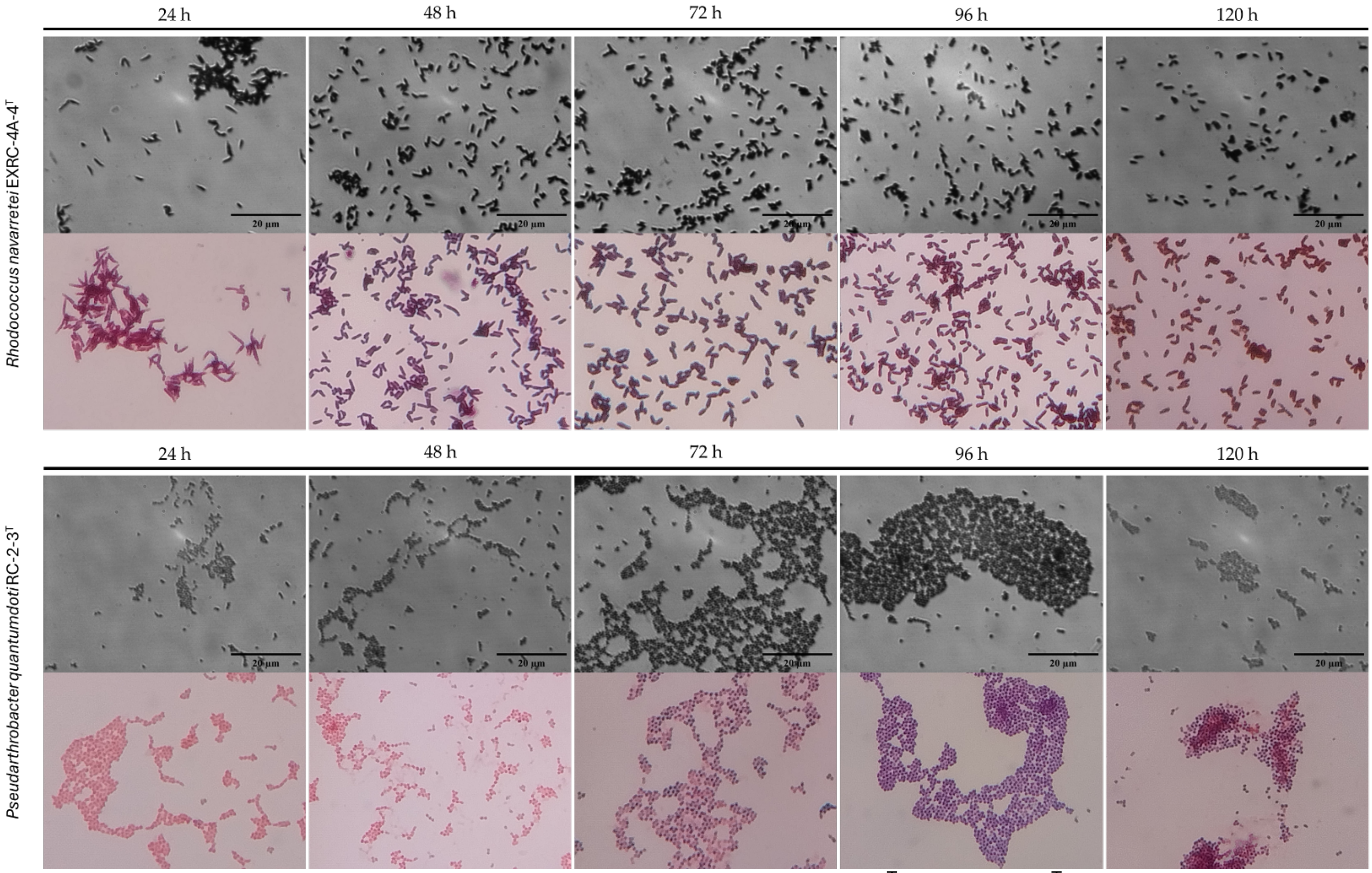

Figure S6. Optical microscopy images of strains EXRC-4A-4^T and RC-2-3^T grown on TSB medium at 28 °C at different times to analyze cell morphology (top row) and Gram-staining (bottom row).
